# Helping Babies Breathe (HBB) training: What happens to knowledge and skills over time?

**DOI:** 10.1186/s12884-016-1141-3

**Published:** 2016-11-22

**Authors:** Akash Bang, Archana Patel, Roopa Bellad, Peter Gisore, Shivaprasad S. Goudar, Fabian Esamai, Edward A. Liechty, Sreelatha Meleth, Norman Goco, Susan Niermeyer, William Keenan, Beena D. Kamath-Rayne, George A. Little, Susan B. Clarke, Victoria A. Flanagan, Sherri Bucher, Manish Jain, Nilofer Mujawar, Vinita Jain, Janet Rukunga, Niranjana Mahantshetti, Sangappa Dhaded, Manisha Bhandankar, Elizabeth M. McClure, Waldemar A. Carlo, Linda L. Wright, Patricia L. Hibberd

**Affiliations:** 1 Mahatma Gandhi Institute of Medical Sciences, Sewagram, India; 2 Lata Medical Research Foundation, Nagpur, India; 3 KLE University’s JN Medical College, Belgaum, India; 4 Moi Teaching and Referral Hospital, Moi University, Eldoret, Kenya; 5 Indiana University School of Medicine, Indianapolis, IN USA; 6 RTI International, Durham, NC USA; 7 University of Colorado School of Medicine, Aurora, CO USA; 8 St. Louis University, St Louis, MO USA; 9 Cincinnati Children’s Hospital Medical Center, Cincinnati, OH USA; 10 Geisel School of Medicine at Dartmouth, Lebanon, NH USA; 11 Children’s Hospital Colorado, Aurora, CO USA; 12 NKP Salve Institute of Medical Sciences, Nagpur, India; 13 Daga Memorial Government Women’s Hospital, Nagpur, India; 14 University of Alabama at Birmingham, Birmingham, AL USA; 15 The Eunice Kennedy Shriver National Institute of Child Health and Human Development (NICHD), Bethesda, MD USA; 16 George Washington University, 5800 Nicholson Lane, #1206, Rockville, MD 20852 USA; 17 Massachusetts General Hospital, Boston, MA USA

**Keywords:** Helping Babies Breathe, Resuscitation, Bag and mask ventilation, Perinatal mortality, Asphyxia, Stillbirth, Training

## Abstract

**Background:**

The first minutes after birth are critical to reducing neonatal mortality. Helping Babies Breathe (HBB) is a simulation-based neonatal resuscitation program for low resource settings. We studied the impact of initial HBB training followed by refresher training on the knowledge and skills of the birth attendants in facilities.

**Methods:**

We conducted HBB trainings in 71 facilities in the NICHD Global Network research sites (Nagpur and Belgaum, India and Eldoret, Kenya), with a 6:1 ratio of facility trainees to Master Trainers (MT). Because of staff turnover, some birth attendants (BA) were trained as they joined the delivery room staff, after the initial training was completed (catch-up initial training). We compared pass rates for skills and knowledge pre- and post- initial HBB training and following refresher training among active BAs. An Objective Structured Clinical Examination (OSCE) B tested resuscitation skill retention by comparing post-initial training performance with pre-refresher training performance. We identified factors associated with loss of skills in pre-refresher training performance using multivariable logistic regression analysis. Daily bag and mask ventilation practice, equipment checks and supportive supervision were stressed as part of training.

**Results:**

One hundred five MT (1.6 MT per facility) conducted initial and refresher HBB trainings for 835 BAs; 76% had no prior resuscitation training. Initial training improved knowledge and skills: the pass percentage for knowledge tests improved from 74 to 99% (*p* < 0.001). Only 5% could ventilate a newborn mannequin correctly before initial training but 97% passed the post-initial ventilation training test (*p* < 0.0001) and 99% passed the OSCE B resuscitation evaluation. During pre-refresher training evaluation, a mean of 6.7 (SD 2.49) months after the initial training, 99% passed the knowledge test, but the successful completion rate fell to 81% for the OSCE B resuscitation skills test. Characteristics associated with deterioration of resuscitation skills were BAs from tertiary care facilities, no prior resuscitation training, and the timing of training (initial vs. catch-up training).

**Conclusions:**

HBB training significantly improved neonatal resuscitation knowledge and skills. However, skills declined more than knowledge over time. Ongoing skills practice and monitoring, more frequent retesting, and refresher trainings are needed to maintain neonatal resuscitation skills.

**Trial registration:**

ClinicalTrials.gov Identifier: NCT01681017; 04 September 2012, retrospectively registered.

**Electronic supplementary material:**

The online version of this article (doi:10.1186/s12884-016-1141-3) contains supplementary material, which is available to authorized users.

## Background

Millennium Development Goal 4 called for a two-thirds reduction in mortality of children under 5 years of age from 1990 rates by 2015 [1]. Between 1990 and 2013 under five mortality decreased from 12.7 to 6.3 million annual deaths, but the Millennium Development Goal was not met because the neonatal mortality rate decreased by only 40%, from 4.7 to 2.8 million, during this same time period. Because the decline in neonatal mortality was slower than the decline in post neonatal mortality, neonatal mortality represents an increasing proportion of the under five deaths (~45%). At current rates, it will be over 150 years before African newborns have the same chance of survival as a baby born today in Europe or North America [2]. Hence, attention is increasingly focusing on reducing neonatal mortality in order to achieve sustainable progress toward future global goals [2–6].

The three leading causes of neonatal mortality worldwide are prematurity (36%), birth asphyxia (23%), and infections (23%) [3]. Neonates who survive birth asphyxia may have such long-term consequences as cerebral palsy, epilepsy, and learning disabilities [7]. An additional 1.2 million intrapartum stillbirths are not included in neonatal mortality rates [3]. Neonatal resuscitation has the potential to prevent perinatal mortality caused by intrapartum related asphyxia for almost two million babies annually [3]. However to be successful, birth attendants (BAs) must be trained to perform appropriate and adequate neonatal resuscitation in the critical first minutes after birth.

The Neonatal Resuscitation Program (NRP) has been the standard of care for resuscitating newborns since 1987 [8, 9]. First Breath, the first controlled resuscitation trial, used simplified versions of NRP and essential newborn care to train BAs in low and middle income countries and demonstrated that essential newborn care, including resuscitation training, significantly reduced still births without increasing the early neonatal mortality rate [10]. Subsequently the American Academy of Pediatrics (AAP), in collaboration with global partners, including Laerdal Medical and *The Eunice Kennedy Shriver* National Institute of Child Health and Human Development (NICHD) Global Network for Women’s and Children’s Health Research (Global Network), developed Helping Babies Breathe (HBB), a simulation-based curriculum to train facility BAs in resuscitation, in resource limited settings [11–14].

HBB focuses on the initial steps of resuscitation, including immediate drying of the baby, providing warmth and additional stimulation to breathe, followed by bag and mask ventilation (BMV) if needed, within the first 60 seconds after birth (The Golden Minute^TM^). HBB training materials use multiple approaches (color, graphic icons outlining three simple care paths, and illustrations depicting the key elements of skills); draw attention to critical decision points; and stress the need to initiate ventilation no later than the end of the first minute after birth [15]. Teaching materials include a Learner Workbook, Facilitator Flip Chart, a neonatal simulator (NeoNatalie^TM^) that allows trainers to manipulate cardinal evaluation signs (crying, breathing, heart rate), and an Action Plan that uses these evaluation signs to guide decision-making and management of the newborn who may range from a healthy wailing newborn to one who needs extra attention before crying and breathing well, or one who needs BMV and advanced care.

Cohort studies suggest that BAs at various skill levels [14, 16–24] can be trained to effectively resuscitate newborns using the HBB methods; however, less is known about the durability of knowledge and skills and the need for re-training [14, 16, 20, 23]. We recently conducted a study of the effect of HBB training on perinatal survival in three sites of the NICHD Global Network, two sites in India and one in western Kenya [25]. The primary outcomes of the study have already been published [26]. Here we evaluate the effect of HBB training on neonatal resuscitation skills and knowledge, as well as retention of knowledge and skills by physicians and nurses who attended deliveries in facilities. The objectives were to evaluate (1) baseline knowledge and skills of BAs; (2) change in knowledge and skills after HBB training; (3) retention of skills and knowledge until refresher training; (4) the effect of refresher training on knowledge and skills of the BAs: and (5) factors associated with loss of skills before refresher training.

## Methods

This study was conducted in Global Network sites in Belgaum and Nagpur, India, and Eldoret, Kenya, areas covered by a prospective, population-based registry which was established in 2008 and included all pregnancy and neonatal outcomes through 42 days postpartum in defined geographic catchment areas. The training intervention was delivered in selected health facilities that provided 24-h coverage for deliveries 7 days/week, served a population that had a minimum perinatal mortality rate of 30 per 1000 registry deliveries in the pre-study period, and delivered 40% of the total registry births in the three sites. The study protocol detailing the design was published previously [25].

### HBB training

The intervention consisted of the following: (1) planning phase; (2) selection of facility staff to be trained as facility-level MTs and identification of all BAs in the participating facilities; (3) rapid scale up of HBB training done in three stages: (a) training of facility-level MTs (b) facility-level training of BAs, (c) refresher training of active BAs (defined as those who regularly attended deliveries); and (4) introduction of a multi-faceted monitoring program soon after the facility-level trainings.

1. Planning Phase
  The goal of the planning phase was to develop and implement an optimal HBB training curriculum for two types of trainees—the facility-level MTs and the facility BAs. The Global Network team worked with the central AAP MT group to develop a two-level HBB training curriculum based on the lessons learned from the HBB training programs to date. Concurrently each of the three study sites identified three HBB country MTs who were experienced in HBB training methodology. These country MTs and the central AAP MTs were a mix of female and male physicians and nurses. Together they trained a select group of new facility MTs who then would train facility BAs.
  For all trainings, we followed the HBB training guidelines, a package designed for adult trainees from different backgrounds, knowledge, professional training and skills, with minimal modifications [15, 27]. The interactive program used multiple learning methods, including self-study/learning, short verbal explanations, demonstrations, practicing in pairs, practical exercises, and group discussions. All training sessions followed the core steps of course preparation and delivery: assembling the teaching materials, distributing learner workbooks in advance, preparing content and teaching methods for each learning group, preparing the classroom space, engaging the learners and evaluating the learners and the course. In addition, trainers provided ongoing monitoring and continued learning in the clinical setting. Detailed agendas of the three training sessions are provided (Additional file 1).
2. Selection of Facility-Level MTs and BAs
  The facility-level MTs at each health facility were pediatricians, obstetricians, anesthetists, general physicians, and staff nurses with adult education experience or an aptitude for teaching who regularly attended deliveries and had expertise in neonatal resuscitation. The initial facility-level BA training included all providers from pediatrics, obstetrics, anesthesia, and nursing departments, as well as facility administrators. However, refresher training was restricted to those providers who had received initial HBB training and attended deliveries regularly (active BAs), based on delivery logs at each facility. The baseline characteristics of BAs were recorded at both initial and refresher training to help evaluate the initial skill level and experience, as well as skill retention at refresher training; these characteristics included age, type of health provider (physicians or nurses), health facility level (primary, secondary, or tertiary), prior resuscitation training (HBB, NRP, NSSK, essential newborn care or others), the average monthly number of births attended and, at refresher training, the average monthly number of births attended in the prior six months, and the interval between initial and refresher training. The general and specialist doctors were categorized as physicians and the trained nurses, nurse midwives, auxiliary nurse midwives were categorized as nurses. Health facilities were classified as primary level if they did not perform Caesarean sections (C-sections), secondary level if C-section staff was available on call, and tertiary level when C-sections were available 24 h a day.
3. Rapid Scale Up of HBB Training
  - **Country-level training of facility MTs:** As above, a group of three experienced central AAP MTs were paired with three experienced MTs at each of the participating Global Network sites to form a female–male, multi-disciplinary (physicians and nurses) country team of MTs to conduct a four-day training of the new facility MTs. The goals of this training of trainers were to rapidly scale up HBB training by training at least one MT per facility (vs. a training cascade), to preserve the integrity of the intervention and to minimize time and training costs. The training agenda was developed in collaboration with the AAP, based on lessons learned to date, including the need for trainers to do pre-training preparation by phone and in person and through post-training evaluation meetings. Each country MT trained a team of six new multidisciplinary facility MTs. The first two days were dedicated to hands-on learning of HBB. During the last two days, the new facility MTs taught small segments of HBB to their fellow team members and developed plans for rolling out training, as the central AAP MTs and country MTs monitored their teaching skills. A roving monitor observed whether the training groups were functioning well and provided feedback after consultations with the team members. Whenever needed, the new facility MT was provided an opportunity to improve his/her skills and extra training was provided until every trainee MT was proficient in resuscitation training. The unique features of these training sessions are summarized in Table 1. The MTs at each facility also facilitated ongoing monitoring and continued learning opportunities in their clinical settings, e.g., participated in monitoring activities such as regular observation of deliveries, resuscitation debriefing.

**Table 1:** Unique features of the Global Network HBB training

• Collaboration with AAP to develop training agendas for 4-day MT and 3-day BA training based on lessons learned to date • Country level training of Facility MTs in May 2012 paired AAP MTs with experienced local MTs at the 3 Global Network sites in an intensive 4-day hands on HBB training of trainers (ToT) that was designed to - Train a large number of Facility MTs to minimize the training cascade and provide at least one MT per facility; - Rapidly rollout trainings in all facilities simultaneously; - Assist MTs in planning effective training of the large number of BAs in the participating facilities • Training involved: - A maximum ratio of 6 trainees:1 trainer; - Maximal opportunities to practice in trainee groups of 6; - Before and after formal testing of knowledge and skills; - Individual sessions with the trainers. • This MT training (ToT) included roving monitors and frequent consultation with the core group and group discussions with the new MTs to ensure that they were engaged and receiving positive feedback. • The new MTs provided 3-day HBB training to their facility BAs between May and September 2012 with mixed groups of physician and nurse BAs. As in the 4-day MT training sessions, this 3-day facility level training of BAs had no didactic sessions; the goal was to provide maximal hands on skill training and open discussion to ensure that every BA could effectively resuscitate newborns. • Testing before and after training was done with standard HBB tools to test resuscitation knowledge and skills, including the standard HBB Knowledge Check (written multiple choice questionnaire), BMV skills test, Objective Structured Clinical Examination (OSCE A), and a case scenario requiring BMV (OSCE B).	ᅟ
  - **Facility-level training of BAs**: The study provided sufficient equipment for facility delivery rooms and resuscitation practice corners. The facility BAs were trained as HBB teams in three-day on-site facility courses by the newly-trained site MTs at their respective facilities between June and October 2012. This course included pre- and post-tests for all participants. Unlike the facility MT training, the primary focus of the BA courses was on training to optimally perform delivery room resuscitation. The BA trainees were taught to prepare for every birth; to focus on immediate, thorough, and vigorous drying of every newborn; to provide routine care (provision of warmth, delayed cord cutting between 1 and 3 min, and initiation of breastfeeding) for babies that breathed spontaneously; to provide more vigorous stimulation and, if needed, suction of mouth and nose, for babies that did not breathe well after drying; and finally to initiate and establish early and effective ventilation within The Golden Minute^TM^ for all babies that did not breathe after the above initial steps. Rather than didactic sessions, skill training and open discussion were provided (in the local language as required) to ensure that every BA could effectively resuscitate newborns. BAs were instructed to resuscitate every non-macerated birth, including those that might previously have been considered fresh stillbirths. High-quality HBB teaching materials and methods were used throughout the training. The HBB teaching materials included a neonatal simulator that felt and weighed like a newborn which could be manipulated by the MT to demonstrate the presence or absence of chest rise, cry and cord pulsations (Laerdal NeoNatalie^TM^); a Learner Workbook for each participant; a large Action Plan poster mounted near each group of six participants that pictured the color-coded paths for the three resuscitation outcomes; and a Facilitator Flipchart that guided teaching and discussions with pictures for the BA on the front and learning content for the MT on the back of each page. The teaching methods included demonstrations of each resuscitation step by the MTs, followed by individual and paired practice to encourage peer teaching and paired learning and standardized case scenarios and exercises for problem solving. This group training included a maximum ratio of six BAs per MT. Because all sites experienced staff turnover, newly-hired BAs were trained by the facility MTs in catch-up individual training or small group sessions, using the same materials and approach as in the initial training sessions.
  - **Refresher training:** A half-day refresher training course for all “active” BAs was prospectively planned for approximately six months after the initial training course. If required to accommodate the staff schedules and clinical duties, multiple refresher training sessions were conducted in each participating health facility. The refresher training included a group review of the entire resuscitation algorithm and demonstration of critical skills through video clips developed by AAP, along with instructional narration by the MTs in local languages as required [28]. The standard facilitator video script was used as a guide to maintain the uniformity of this instructional narration [29]. This was followed by practice in pairs and discussions among the BAs. Lastly, the BAs discussed the details of various monitoring activities—the need for each monitoring activity, the benefits, challenges and successful solutions. As with the initial training, the refresher training included pre- and post-tests to assess retention of the content.
4. Monitoring Activities
  Monitoring activities were introduced immediately after the initial facility-level training of BAs and carried out as a part of continuation of learning/training techniques best described as supportive supervision [25, 30]. The study staff at each facility monitored daily BMV practice sessions when BAs reported for work and signed the logbooks; daily BA checks of availability, cleanliness, and function of resuscitation equipment; regular observation of deliveries; debriefing after every resuscitation; and audits of every perinatal death on a continuous ongoing basis. Additionally, once a month, the MTs made a one full-day quality assurance (QA) visit to observe HBB skills directly during deliveries or test the BAs using a neonatal simulator if no deliveries were available.
  Information from all the participating facilities was compiled by the site HBB coordinators. This included compliance with monitoring activities, details of each resuscitation event in each of the participating facilities, and the outcomes, using a standard format. Calls were conducted every two weeks between the central staff (NICHD and RTI International) and each site coordinator to discuss individually every baby that had required resuscitation in the 72 participating facilities, including confirming whether each live born baby (non-macerated) was immediately dried, whether the baby cried or breathed spontaneously after drying, required additional stimulation, whether the baby received BMV, and whether the baby was alive or dead (fresh stillbirth) at discharge from the delivery room (Additional file 2)—data required for standard newborn indicator metrics. Quantitative practice data were not recorded, but physicians were described as less compliant with daily BMV practice than nurses. After the biweekly calls, the site coordinators discussed the content of the calls with their facility staff to close the monitoring loop. The facility staff were encouraged to track their own progress as well, e.g., proportion of BAs performing daily practice; the number of macerated stillbirths (for whom resuscitation was not required); and the percent of non-macerated births that did not cry at birth, responded to additional stimulation after drying, required BMV, and survived until discharge from the delivery room. A monthly consolidated report from the data coordinating center at RTI International included compliance rates of individual facilities with each of the four monitoring parameters- one QA visit every month, one unannounced visit every three months, resuscitation debriefings done for at least 90% of babies not crying at birth, and death audits done for at least 90% of perinatal deaths.

### Evaluation of knowledge and skills

The knowledge evaluation of the BA trainees was done using the standard HBB Knowledge Check before and after (pre-post scores) initial and refresher training. The Knowledge Check was a 17-item written multiple choice questionnaire [31]. The trainee was expected to answer 14 out of 17 of the questions correctly to successfully complete the written knowledge evaluation.

The skills evaluation was done with the neonatal simulator and included ability to perform BMV effectively (appropriate chest rise and ventilation rate) and successful completion of the Objective Structured Clinical Evaluation (OSCE) A and OSCE B. These evaluations were performed according to study protocol to minimize potential bias and inter-rater variability. The Facilitator Flip Chart provided detailed instructions for administering the three skill tests i.e. BMV, OSCE A and OSCE B. Every trainee was required to successfully complete the BMV Performance Evaluation (seven out of seven steps) before attempting the OSCE evaluations [32]. OSCEs were developed from the NRP megacode (validated by Lockyer et al. [33]) and required an 80% correct performance with a small number of critical elements required to “pass.” OSCE A tested the skills and decision-making in routine care and the initial steps of The Golden Minute^TM^ [34]. The trainees were expected to correctly perform three key steps with the simulator (dries thoroughly, recognizes baby is not crying, and positions head and clears airway) and perform a total of 10 out of 13 steps to successfully complete OSCE A. OSCE B was a comprehensive skill testing that included elements of OSCE A, the skills of BMV and additional decision making based on assessment of heart rate [34]. Trainees were expected to correctly perform four key steps (recognizes baby is not breathing, ventilates at 40 breaths per minute, looks for chest movement, and performs the five steps to improve ventilation) and perform a total of 14 out of 18 steps correctly to successfully complete OSCE B. OSCE A and OSCE B were not tested before the initial training as trainees were not expected to have these skills prior to their training. After the initial training, the trainees received positive supervision and monitoring to assist in the retention and mastery of the knowledge and skills of HBB during daily practice, site visits and deliveries. Therefore trainees’ skills were evaluated using the comprehensive OSCE B just prior to and after the refresher training. Those who passed OSCE B after initial training and failed the evaluation prior to the refresher training were considered to have a decline in OSCE B skills.

### Data management

The study data were recorded on paper forms and then entered into a password protected electronic data base by study staff at each site. Data were securely transmitted on a regular basis to the central data center. Data entry software included consistency checks, with cross-form edits performed centrally at the data center and resolved locally. Each site performed local data review and edits.

### Statistical analysis

Chi-square tests of proportion were used to test differences in pass rates between the types of health providers. McNemar’s test was used for paired comparisons to test the proportion of BAs who passed the MCQ after initial training but failed it in the pre-refresher training. A logistic regression model was used to assess the association between BA characteristics and loss of skills. Those that were significant (*p* < 0.05) in univariate models were retained as predictors in the final model. The variables in the final model were type of training (group vs. individual), type of health facility (primary, secondary, or tertiary), type of health provider (physician, nurse), exposure to prior resuscitation training, number of births attended per month in the last six months (based on recall), study site, and age of the BAs. The dependent variable was the probability of failing the pre-refresher OSCE B test after passing the post-initial OSCE B. Unadjusted odds ratios (ORs) and 95% confidence intervals (CIs) were estimated by fitting univariate models with each of the predictors. Adjusted ORs and 95% CIs were estimated by fitting a multi-variable logistic regression model with all of the predictors included.

### Ethical considerations

The HBB study protocol was approved by the institutional review committees of all the participating sites, partner institutions, and the Indian Council of Medical Research. A Data Monitoring Committee appointed by NICHD reviewed the study protocol and progress. All the MTs and BAs participating in the training signed the approved written informed consent form that informed them about the study including the testing of knowledge and skills.

## Results

**Fig. 1:**
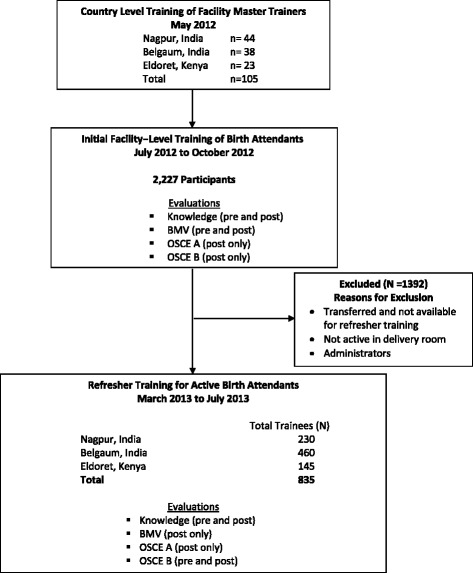
CONSORT flow diagram of the Global Network HBB training study

The 105 MTs trained 2227 BAs in the HBB curriculum during 236 workshops (Fig. 1) from June to October 2012. 835 active BAs (35% physicians, 65% nurses) received refresher training and were included in the analysis of knowledge and skills over time. The characteristics of these 835 active BAs are shown in Table 2: 17%, 28% and 55% were from Eldoret, Nagpur and Belgaum sites respectively. 76% of the BAs across all three sites reported no prior resuscitation training before HBB training. At refresher training, 47% estimated that they delivered between 11 and 40 babies/month and 19% estimated that they delivered more than 40 babies per month. One third of BAs in Belgaum and Kenya, as compared to only 8% in Nagpur, were trained later through catch-up training, after the initial group training, reflecting higher BA turnover in Belgaum and Kenya. The mean (SD) time interval in months between the initial and refresher trainings for these catch-up trainees was significantly less than that for the initial cohort of trainees (3.8 ± 2.9 vs. 7.8 ± 0.9, *p* < 0.0001). Of the 291 physicians who participated in refresher training, 130 (45%) had received their initial training as catch-up training. All these physicians served in primary care facilities. Among nurse BAs, 99 (18%) had received their initial training as catch-up training. Of these nurse BAs, 98% were in secondary level facilities and 2% were in tertiary facilities.

**Table 2:** Characteristics of Active Birth Attendants^a^

	Belgaum (*n* = 460)	Nagpur (*n* = 230)	Kenya (*n* = 145)	Total (*n* = 835)
Birth attendants trained, n (%)				
Initial Group training	291 (63)	211 (92)	104 (72)	606 (73)
Catch-up Individual training	169 (37)	19 (8)	41 (28)	229 (27)
Facility level^b^, n (%)				
Primary level facilities	42 (9)	25 (11)	113 (78)	180 (22)
Secondary level facilities	180 (39)	51 (23)	32 (22)	263 (32)
Tertiary level facilities	235 (51)	149 (66)	0	384 (46)
Health provider n (%)				
Physician	214 (46)	77 (34)	0	291 (35)
Nurses	246 (54)	153 (66)	145 (100)	544 (65)
Prior resuscitation training, n (%)	118 (27)	47 (20)	38 (26)	203 (24)
No prior training	342 (73)	183 (80)	107 (74)	632 (76)
Physicians	195 (57)	57 (31)	0	252 (40)
Nurses	147 (43)	126 (69)	107 (100)	380 (60)
One prior training	93 (79)	45 (96)	35 (92)	173 (85)
Two or more prior trainings	25 (21)	2 (4)	3 (8)	30 (15)
Number of births attended per month, n (%)				
1–4	18 (4)	7 (4)	17(12)	42(6)
5–10	119 (28)	46 (27)	47(31)	212(29)
11–20	124 (29)	39 (23)	37(26)	200(27)
21–40	85 (20)	40 (23)	20(14)	145(20)
> 40	83 (19)	39 (23)	19(14)	141(19)
Birth Attendants Age Mean (SD)				
Physician	26 (7)	31 (8)	-	27 (5)
Nurse	30 (8)	36 (11)	39 (10)	34 (10)
Age Quartiles n (%)				
≤ 24 years (Lower Quartile)	178 (39)	29 (13)	9 (6)	216 (26)
25–38 years (Inter Quartile)	227 (49)	132 (57)	65 (45)	424 (51)
> 39 years (Upper Quartile)	55(12)	69 (30)	71 (49)	195(23)

^a^Active Birth Attendants were defined as BAs who received both the initial and refresher training

^b^Levels of facilities- Primary = No Caesarian section (C-sections) performed, Secondary = C-section staff available on call, Tertiary = C-sections available 24 h a day

All BAs from the Kenya site were nurse midwives from either primary level facilities (no C-sections performed) or secondary level facilities (C-section staff available on call). In contrast, many of the BAs in Nagpur and Belgaum were physicians; deliveries in Nagpur were predominantly in tertiary level facilities where C-sections were available 24 h a day.

Table 3 shows the proportion of BAs with a “passing” knowledge score (by multiple choice testing) before and after the initial HBB and refresher trainings. Initial HBB training improved knowledge significantly: 99% of the trainees passed the knowledge test post-training compared to 74% who passed it pre-training (*p* < 0.0001). Physicians were significantly more likely than nurses to have a passing knowledge score before the initial HBB training (86% vs 68%, *p* < 0.0001), but, after the initial HBB training, all physicians and 98% nurses passed the post-training knowledge exam.

**Table 3:** Training Outcomes for Active Birth Attendants—Initial and Refresher Trainings

	Initial training (*N* = 835)			Refresher training (*N* = 835)			Change over time
	Pre	Post	pre vs post *P* value	Pre	Post	pre vs post *P* value	Initial Post vs. Refresher Pre *P* value
Passed n (%)							
Knowledge	618/835 (74.0)	826/835 (98.9)	<0.0001	822/828 (99.3)	822/828(99.3)	NE^b^	0.43
Physician	249/291 (85.5)	291/291 (100)	NE^b^	291/291 (100)	291/291(100)	NS^a^	NE^b^
Nurses	369/544 (67.8)	535/544 (98.3)	<0.0001	531/537 (98.8)	531/537(98.8)	NS^a^	0.43
Physicians vs. nurses, *P* value	*p* <0.0001	*p* = 0.03	-	*P* = (0.07)	*P* = (0.005)	-	-
Bag and mask skills	39/833 (4.6)	809/833 (97.1)	<0.0001	-	802/835(97.2))	-	-
Physician	10/291 (3.4)	291/291 (100)	NE^b^	-	291/291(100	-	-
Nurses	29/542 (5.3)	518/542 (95.6)	<0.0001	-	511/534(95.7)	-	-
Physicians vs. nurses, *P* value	0.21	0.0003	-	-	0.0003	-	-
OSCE station A	-	830/835 (99.4)	-	-	825/835(99.8)	-	-
Physician	-	291/291 (100.0)	-	-	291/291(100.0)	-	-
Nurses	-	539/544 (99.0)	-	-	534/544(99.8)	-	-
Physicians vs. nurses, *P* value	-	0.10	-	-	0.46	-	-
OSCE B	-	781/786 (99.4)	-	576/707 (81.4)	709/715 (99.0)	<0.0001	<0.0001
Physician	-	275/275 (100.0)	-	190/252 (75.4)	253/254 (99.6)	<0.0001	NE^b^
Nurses	-	506/511 (99.0)	-	386/455 (84.4)	456/461 (98.9)	<0.0001	<0.0001
Physicians vs. nurses, *P* value	-	0.09	-	0.002	0.32	-	-

^a^NS = Not significant

^b^NE = Not Estimable

*P*-values were not estimable for the tests where no physician failed

Improvements in resuscitation skills after training were more impressive than the gains in knowledge, in part because BMV skills were so low initially. Prior to the initial HBB training, only 4.6% of BAs (3% of physicians and 5% of nurses) could ventilate a newborn mannequin effectively, as measured by the straightforward BMV skills test, but all physicians and 96% of nurses passed the test after initial training (*p* < 0.0001 pre- vs. post-initial training). OSCE A was assessed after initial and refresher trainings with 99.4 and 99.8% of BAs passing the test at both time points, respectively.

Table 3 also shows the proportion of BAs passing the OSCE B skills test post-initial training (99%). Prior to refresher training, the proportion passing fell to 81% (*p* < 0.0001), but after refresher training 99% passed the OSCE B. Physicians were less likely than nurses to retain OSCE B skills pre-refresher training (*p* < 0.002). The interval from initial to refresher training for those who failed the pre-refresher OSCE B after passing it in post-initial assessment was not longer than for those that passed both (mean [SD], 6.9 [2.8] vs 6.5 [2.5] months, *p* = 0.1).

Table 4 shows factors associated with deterioration of OSCE B skills. 25% of physicians and 16% of nurses (*p* = 0.002) failed pre-refresher training OSCE B. Among the seven characteristics in the regression model, facility type (p < 0.0001) and prior training (*p* < 0.01) were the two significant predictors of deterioration of OSCE B skills. BAs from tertiary facilities (adjusted OR 8.83; 95% CI 2.91, 26.78) and those with no prior resuscitation training (adjusted OR 2.56; 95% CI 1.28, 5.11) were most likely to fail the pre-refresher training OSCE B. However, those who received their initial training during catch-up rounds in individual or small-group training (adjusted OR 0.497; 95% CI 0.28, 0.88); and the BAs from Nagpur site (adjusted OR 0.154; 95% CI 0.05, 0.50) were less likely to lose the skills over time.

**Table 4:** Factors associated with loss of resuscitation skills as assessed by (OSCE B) at the time of refresher training

	Passed Post Initial Training OSCE-B			
	Failed Pre-Refresher Training OSCE-B (125, 19 %)	Passed Pre-Refresher Training OSCE-B (550, 81 %)	OR (95 % CI)	Adjusted OR (95 % CI)
Birth Attendants Trained, n (%)	125 (19)	550 (81)		
Initial Group Training	79 (17)	382 (83)	1.0	1.0
Catch-up training	46 (21)	168 (79)	1.324 (0.882.1.988)	0.497 (0.282, 0.877)
Facility Level, n (%)				
Primary level facilities	17 (12)	124 (88)	1	1
Secondary level facilities	20 (8)	197 (92)	0.741 (0.374, 1.468)	0.920 (0.354, 2.391)
Tertiary level facilities	88 (28)	224 (72)	2.866 (1.631, 5.035)	8.834 (2.914, 26.784)
Health provider, n (%)				
Physician	61 (25)	181 (75)	1.0	1.0
Nurses	64 (14)	369 (86)	0.515 (0.347, 0.763)	1.058 (0.611, 1.831)
Prior resuscitation training, n (%)				
No prior training	112 (22)	400 (78)	3.230 (1.765,5.909)	2.560 (1.283, 5.110)
Prior resuscitation training1	13 (8)	150 (92)	1	1
Number of births attended per month, n (%)				
1–4	7 (19)	29 (81)	0.611 (0.243,1.535)	1.044 (0.382, 2.857)
5–10	30 (17)	145 (83)	0.524 (0.297, 0.924)	0.888 (0.475, 1.659)
11–20	27 (16)	146 (84)	0.468 (0.262,0.836))	0.668 (0.356, 1.254)
21–40	21 (18)	93 (82)	0.572 (0.306,1.07)	0.813 (0.411, 1.606)
> 40	32 (28)	81 (72)	1.0	1.0
Site, n (%)				
Belgaum	89 (21)	327 (79)	1.583 (0.905,2.613)	0.527 (0.191, 1.452)
Nagpur	16 (13)	110 (87)	0.822 (0.405.1.668)	0.154 (0.047, 0.502)
Kenya	20 (15)	113 (85)	1	1
Age category				
Age < =24 years	44 (22)	155 (78)	1.481 (0.848,2,587)	0.508 (0.299, 1.126)
25–38 years	58 (17)	275 (83)	1.100 (0.649,1.866)	0.632 (0.328,1.126)
> =39 years	23 (16)	120 (84)	1	1

Footnote: The figures in parentheses in the first two columns indicate percentage from the total trainees with that risk factor, i.e. considering row total as 100

## Discussion

Our study is the first large multi-national pre-post study of the impact of initial HBB training, followed by refresher training, on resuscitation knowledge and skills over time, using a state-of-the-art curriculum, standardized training protocol and evaluation methods, and ongoing supportive supervision and monitoring activities. Our goal was to address the methodologic problems of previous research in resuscitation training [35].

One of the most striking findings of our study of a large cohort of 835 active BAs trained and retrained in the HBB curriculum in 71 facilities in India and Kenya was that 76% of active BAs reported no prior training in neonatal resuscitation, despite efforts in India, Africa and other low and middle income countries to encourage facility deliveries and the relatively high BA delivery rates (47% of the BAs delivered between 11 and 40 babies per month and 19% delivered more than 40 babies per month.

The lack of resuscitation training was evidenced by a large initial knowledge-skills gap among the BAs: at the initial HBB training, 74% of BAs passed the pre-training knowledge assessment but only 5% were able to demonstrate effective ventilation of the neonatal mannequin. Our findings are consistent with Singhal et al., who also found a low concordance between knowledge and skills [13]. A majority of their trainees in Kenya and Pakistan required additional practice before they could demonstrate mastery of BMV skills at post-training assessment. Successful neonatal resuscitation requires rapid assessment, prompt decision making, and immediate action. It is a composite of adequate knowledge related to basic resuscitation, good technical skills, and the ability to synthesize these two domains into a complex behavior pattern, in addition to ongoing practice to maintain these complex skills. To achieve this end, we trained a large number of MTs (an average of 1.6 per facility) and BAs using four-day MT and three-day BA small-group training workshops with hands-on sessions and generous opportunities for individuals and pairs of BAs to practice resuscitation skills and lead skill sessions for the group. The combination of this unique training and sufficient equipment resulted in nearly 100% of BAs successfully passing knowledge and skills tests after the initial HBB training. The significant difference in pre-training knowledge and ventilation tests between physicians and nurses (*p* < 0.0001) disappeared after HBB training when more than 98% of both doctors and nurses passed the knowledge tests and the proportion of both nurses and doctors who correctly ventilated a neonatal model increased from approximately 4% to 97%.

Our study provided sufficient ventilation equipment for facility delivery rooms, resuscitation practice corners, and adequate numbers of MTs to ensure ongoing mentoring and supportive supervision of BAs: daily ventilation practice, equipment checks, death audits, resuscitation debriefings, and announced and unannounced site visits at each facility, as well as unique biweekly site conference calls to review the management of each resuscitated newborn in detail and the outcomes of all births before discharge at each facility.

However, the knowledge-skills gap that was evident before the initial HBB training recurred months later at refresher training despite ongoing mentoring and supportive supervision, when resuscitation skills, as measured by OSCE B, were less likely to be retained than knowledge (81% vs. 99%). At pre-refresher testing, BAs from tertiary facilities had nine times higher risk than those at lower-level facilities of failing the OSCE B, after having passed it at the end of initial training—a surrogate for loss of resuscitation skills. The higher skill-deterioration rate of BAs in tertiary facilities who care for high risk patients was unexpected. It may be due to a combination of factors, including less daily practice, whether due to busy schedules, timing of resident rotation, understaffing, increased workload or even a higher proportion of physicians in tertiary-level facilities, resulting in a less frequent BMV practice. During the biweekly reviews, the qualitative reports suggested that physicians were less compliant to daily BMV practice than the nurses, with assertions that they were ‘too busy” or “not in the delivery area.” Lack of previous resuscitation training prior to HBB training was associated with a 2.5 times greater risk of pre-refresher OSCE B failure. This may mean that all or a subset of BAs need repeated trainings to decrease the risk of failure. Those whose initial training was late (catch-up training) were less prone to skill deterioration; the mean time between initial and refresher trainings was also almost half compared to the initially trained BA cohort (3.8 ± 2.9 months vs. 7.8 ± 0.9 months, *p* < 0.0001), suggesting that recent training may be associated with improved skill performance. The lack of information on the optimal interval for training was recently noted in the Part 7 2015 Neonatal Resuscitation Consensus document: no evidence-based, effective strategy for teaching, assessing, and maintaining resuscitation knowledge and skills has been identified and that the existing evidence is of low quality [35]. Hands-on simulation training is widely accepted as the best available model for teaching complex behavior; however, the published evidence to date is of very low quality and there is no published evidence for improvement in patient outcome. Despite the lack of evidence for an optimal retraining interval, refresher training at least once a year was recommended to maintain skills.

## Conclusions

Our large, rigorous pre-post cohort training study documented that facility BAs in 71 facilities in India and Kenya improved their knowledge and neonatal resuscitation skills after HBB training by MTs and supportive supervision during active monitoring and evaluation over the subsequent year. That 18% of BAs failed pre-refresher training skills tests suggests the need to identify such BAs earlier in order to provide the individualized training, supervision, and practice necessary to ensure that their resuscitation skills are durable over time or to reassign them to a less critical care area. The 18% failure rate should also prompt future researchers to compare different time intervals, structures or content of daily practice or different supervisory models to improve resuscitation skill retention. The risk factors for deterioration of resuscitation skills also suggest that resuscitation training should take place earlier and that administrators should ensure that staffing patterns in tertiary facilities with high delivery volume provide adequate time, motivation and accountability to maintain BA resuscitation skills.

As the HBB program paves the way for its successor, Helping Babies Survive, which includes the HBB 2nd edition and care of a newborn after immediate delivery room resuscitation, it will provide opportunities to incorporate standardized data collection, quality improvement activities, and institutional support for registries [36].

Given the low quality of evidence for simulation training, it is essential that future research addresses the knowledge gaps identified by the recent Wyllie et al. report [35], including different content/structures of daily practice or different supervisory models and documents the critical link between skill training and improved early neonatal survival in different medical and cultural settings.

## Additional files

Additional file 1: Detailed agendas of the Global Network HBB trainings. (DOC 114 kb)
Additional file 2: Biweekly HBB monitoring report format for calls between central and site teams. (XLSX 30 kb)

## Abbreviations

AAP: American Academy of Pediatrics
BA: Birth attendant
BMV: Bag and mask ventilation
CI: Confidence interval
Global Network: The NICHD Global Network for Women’s and Children’s Health Research
HBB: Helping Babies Breathe
MT: Master trainer
NICHD: *The Eunice Kennedy Shriver* National Institute of Child Health and Human Development
NRP: Neonatal Resuscitation Program
NSSK: Navjaat Shishu Suraksha Karyakram- a state run basic neonatal resuscitation training in India
OR: Odds ratio
OSCE: Objective structured clinical evaluation
QA: Quality assurance
SD: Standard deviation
