# Supplementary material for: Helping Babies Breathe (HBB) training: What happens to knowledge and skills over time?
Source: BMC Pregnancy Childbirth. 2016 Nov 22;16:364. doi: 10.1186/s12884-016-1141-3 (PMC5120476; doi:10.1186/s12884-016-1141-3)
Supplement: Additional file 1: — Detailed agendas of the Global Network HBB trainings. (DOC 114 kb) [file 12884_2016_1141_MOESM1_ESM.doc]

**Additional File 1. Sample Training Agendas**

**1A. Agenda for the Country Level Training of Master Trainers**

**1B. Agenda for the Facility Level Training of Birth Attendants**

**1C. Agenda for the Refresher Training of the “active” Birth Attendants**

**Global Network Helping Babies Breathe (HBB) Training**

**1A. COUNTRY LEVEL TRAINING OF MASTER TRAINERS**

|  | **Topics** | **Faculty/staff** |
| --- | --- | --- |
| **Pre-training** | Print materials are distributed (Learner Workbook, Facilitator Flipchart) | Site HBB Staff |
| **Day 0** | **MASTER TRAINER PREPARATION** |  |
| 2-6PM (4 hrs TBD) | **Trainers meet on site**  Hold meeting with local faculty and staff (not trainees) prior to start of course (allow for a 4 hour-long meeting)   - - - Clarify goals for each day of training and course objectives. - Review training agenda in detail: model and review the methods for presentation, demonstration, practice (All faculty annotate their flipcharts with teaching best practices from this dialogue - -e.g. breath-holding exercise in Golden Minute.) - Set up room: rectangular or square tables of 6, 3 learner dyads per table, and seat for facilitator - On table: one facilitator flip chart, 3 NeoNatalies filled with water, sand timer (RTI to provide 1 timer/pair of learners) - Action Plan hanging on walls near tables - Discuss and demonstrate how to use flip charts - Discuss how to use the simulators. - Discuss issues from HBB training - Revised WHO suction guidelines - Emphasize stimulation (rubbing back) - Placement of baby for first steps (on mothers’ abdomen) and for resuscitation (beside mother) - 3 levels of “help” (helper, call for help, seek advanced care) - Feedback and debriefing techniques - Process for administering pretests (MCQ and BMV) and post-tests (MCQ, BMV, OSCE A, and OSCE B) - Site faculty to prepare and present standardized training kits: “Master Trainer Kit” to include local clean birth kit (soap, cord ties/clamps, razor/scalpel, extra blanket) plus pillow or towel to use as mother’s abdomen and bell to call meeting participants (site provides). - Emerging leadership of HBB training at each site. - Identify ‘floater’ for training (trainer who observes tables, ensure consistency among the tables)Site has all forms available and place for notes at each table (develop checklist to run course) - Discuss simulation and debriefing processes | AAP, RTI, NICHD, Site Faculty |
| **DAY 1** | **LEARNER PREPARATION (Provider Component)** |  |
| 7:30 – 9AM | **Arrival, check-in and pre-testing**  Trainees arrive, complete pre-test knowledge check and complete bag and Mask Skill Check with Master Trainers (1 attempt/5 min per person and multiple stations for skill check). | Site Faculty, AAP Faculty, RTI Staff |
| 9 – 10:45AM | **Welcome and introductions**  Opening prayer  Faculty, Staff and Participant Introductions **(40 min)**  *Name and institution, why they are attending the training, role in hospital/health center and any teaching experience*  **Overview of GN protocol and goal of the GN HBB study (15 min)**  **Site introduction of facilities for HBB study (20 min)**  **HBB Objectives (30 min)**  Need for resuscitation in resource-limited countries and overall goal to have person trained at every delivery to help baby breathe. Global perspective to be discussed. (AAP) Regional/local perspective (Local) | Site Faculty (SFI or HBB Program Coordinator)  RTI Staff  Site HBB Program Coordinator  AAP Faculty/Site Faculty |
| 10:45-11:15 | **Break** |  |
| 11:15-11:45 | **Opening Visualization and Orientation to HBB Materials**  Learner Materials overview (clinical and teaching aids):   - - Learner Workbook   - Facilitator Flipchart   - Review the of the Simulator and how to use it: *Emphasis on the non verbal clues generated in the simulator. How to do it, what to expect of the new learner etc. and what is expected of them during this training as well as how to use the simulator in the field.*   - HBB Action Plan | Faculty |
| 11:45-12:30 | **Preparation for Birth**  Preparation for birth: ***Demonstration*** *of exercise to include mother, birth attendant, helper at front of room;* THEN follow Facilitator Flip Chart [page numbers in brackets]  *Table presentation/demonstration, practice of 4 skills* (3 learner dyads per table):   - Identify helper & review emergency plan [3b] - Prepare area for delivery (clean, warm, well lighted) [3b] - Wash hands [3b] - Prepare area for ventilation & check equipment [3b]   Check yourself questions, exercise and small group discussion  Return to large group for summary comments on skills technique and teaching methodology; discuss important questions with whole group; draw conclusions (achieve consensus) from small group discussion. | Faculty |
| 12:30 – 1:15PM | **Routine Care**  *Table presentation/demonstration, practice of 5 skills, and check yourself questions:*   - - Dry thoroughly (if meconium is present, clear airway prior to drying) [4b]   - Evaluation: Is the baby crying? [5b]   - Keep warm (skin-to-skin, cover baby with dry blanket & put on hat), check breathing, cut cord, encourage breastfeeding [6b]   - How to clamp or tie and cut umbilical cord [7b]   *Exercise: Routine care and Group Discussion* [8b]  Return to large group for summary comments on skills technique and teaching methodology; discuss important questions with whole group; draw conclusions (achieve consensus) from small group discussion. | Faculty |
| 1:15 – 2PM | **Lunch** |  |
| 2 – 3:30PM | **The Golden Minute, part I (45min)**  *Table presentation/demonstration, practice, check yourself questions:*   - - Clear airway *if blocked*, stimulate breathing [9b]   - Evaluation: Is baby breathing well? [10b]   *Exercise: The Golden Minute-clear the airway and stimulate breathing and Group Discussion* [11b]  Return to large group for summary comments on skills technique and teaching methodology; discuss important questions with whole group; draw conclusions (achieve consensus) from small group discussion points.  **The Golden Minute, part II (45 min)**  *Table presentation/demonstration, practice, check yourself questions:*   - Ventilate with bag and mask (initiate ventilation) [12b] - How to ventilate with bag and mask (technique) [13b] - Evaluation: Is the baby breathing well? [14b]   *Exercise: The Golden Minute (including initial steps) part II and Group Discussion* [15b]  Return to large group for summary comments on skills technique and teaching methodology; discuss important questions with whole group; draw conclusions (achieve consensus) from small group discussion points. | Faculty |
| 3:30 – 4pm | **Break** |  |
| 4- 5pm | **Continued Ventilation with Normal and Slow Heart Rate**  Table presentation/demonstration, practice, check yourself questions:   - Call for help, Improve ventilation [16b] - Is the heart rate normal or slow? [17b] - Ventilate until the baby is breathing well, then monitor with mother [18b] - Continue ventilation and seek advanced care [19b] - Transport mother and baby together, and support the family [20b]   *Exercise: continued ventilation with normal heart rate and Group Discussion* [21b]  *Exercise: continued ventilation with slow heart rate and Group Discussion* [22b]  Return to large group for summary comments on skills technique and teaching methodology; discuss important questions with whole group; draw conclusions (achieve consensus) from small group discussion points. | Faculty |
| 5 – 5:30PM | **Feedback on written evaluation**  General comments on common themes regarding missed items  **Mastering the Action Plan and Preparing for Evaluation**  Practice and scenario development homework (6 cases p. 37 Learner Workbook)  Overview of Evaluation – Knowledge check, bag and mask skills, OSCE A and B  **Preparing for day 2 – Facilitator/Master Trainer Component** | Faculty |
| **Day 2** | **EVALUATE MASTER TRAINER CANDIDATES and PRACTICE as MASTER TRAINERS (Facilitator/Master Trainer Component)** |  |
| 8-8:30AM | **Welcome, Review of Day and Plan for Day 2** | AAP Faculty |
| 8:30AM – 12:30PM | **HBB Evaluations and Practice Facilitation**  Facilitating the cognitive and performance evaluations   - Knowledge check - Bag and mask skill check - OSCE- A & B *(photocopies of OSCE exams at tables)* Role Play OSCE A as large group, assigning roles to each learner (also provide feedback as a group):   - - 1. Learner being tested       2. Learner ensuring baby dried and crying evaluated       3. Learner ensuring airway cleared and baby stimulated       4. Learner ensuring baby is breathing well by the Golden Minute®       5. Learner ensuring baby is kept warm (skin-to-skin, dry blanket covering baby, hat on)       6. Learner ensuring umbilical cord is clamped or tied; cut between 1-3 minutes - Administer actual OSCE A and B with small groups at tables; each Master Trainer candidate takes the role of the Evaluator after successfully completing the OSCE as the birth attendant with a helper who responds but does not prompt. All members of group participate in debriefing. Faculty member evaluates first candidate only, then guides as necessary. - Master Trainer Faculty demonstrates taking bag apart with dyads practicing at tables | AAP Faculty |
| 12:30- – 1:30PM | **Lunch** |  |
| 1:30 – 4:15PM | **Practicing facilitator/master trainer skills**  **Work in small groups – 10 minutes for each person/activity**   - - Practice presenting flipchart pages (each participant leads demonstration, practice with Action Plan with feedback from facilitator, check yourself ; have group give feedback to master trainer student   - Short presentation on debriefing and Practice leading 6 case scenarios (those developed to fit “Trace six cases”) with practice in dyads and debriefing. | AAP Faculty |
| 4:15 – 4:30PM | **Break** |  |
| 4:30- 5PM | **Debrief as large group:**  What makes a good HBB Facilitator and Master Trainer? (Challenges, successful practices/methods) | AAP Faculty |
| 5-5:30PM | **HBB implementation**  Facilitating a successful course: participants, staff, space, materials  AAP HBB Resources  **Preparation for day 3 – Implementation and Operationalization** | AAP Faculty |
| **Day 3** | **IMPLEMENTATION AND OPERATIONALIZATION** |  |
| 8:15 – 8:30AM | **Review of key points and challenges to facilitating a successful course** |  |
| 8:30 – 9:30AM | **Global Network HBB study implementation**  Data collection  Training timeline and retraining | RTI Faculty |
| 9:30-10:15AM | **Quality assurance and ongoing monitoring activities** | RTI Faculty |
| 10:15-10:30AM | **Break** |  |
| 10:30 -12PM | **Small group individual planning and next steps**   - Implementation cascade at each delivery site - Pairing experienced and novice facilitators: mapping out the training cascade and planning future trainings - QA component: integrating practice and feedback into daily operations - QA monitoring system | Site Faculty |
| 12– 1PM | Report on next steps to entire group | Site Faculty and Participants |
| 1PM | Closing Ceremony with certificates and Photo | All |
| 2 – 4PM | Debrief with study team | AAP, RTI, NICHD, Site Faculty |

**Global Network Helping Babies Breathe (HBB) Training**

**1B. FACILITY LEVEL TRAINING OF BIRTH ATTENDANTS**

|  | **Topics** | **Staff** |
| --- | --- | --- |
| **Pre-training** | Print materials are distributed (Learner Workbook, Facilitator Flipchart) |  |
| **Day 0** | **TRAINING PREPARATION** |  |
| 2-6PM (4 hrs) | - - - Review goals for each day of training and course objectives. - Review training agenda in detail: model and review the methods for presentation, demonstration, practice - Set up room: rectangular or square tables of 6, 3 learner dyads per table, and seat for facilitator - On table: one facilitator flip chart, 3 NeoNatalies filled with water, sand timer - Action Plan hanging on walls near tables - Review use of flip charts - Review use of simulators - Plan process for administering pretests (MCQ and BMV) and post-tests (MCQ, BMV, OSCE A, and OSCE B) |  |
| **DAY 1** | **LEARNER PREPARATION (Provider Component)** |  |
| 8:00 – 9:00AM | **Arrival, check-in and pre-testing***   - Trainees arrive, complete pre-test knowledge check and complete Bag and Mask Skill Check with Master Trainers (1 attempt/5 min per person and multiple stations for skill check - Serve breakfast close to registration and testing area.   ****Note****: Pretesting may be scheduled on Day 0, shifting welcome and introductions as the first activity for Day 1* |  |
| 9 – 9:20AM | **Welcome and introductions**  Opening prayer – dedication and prayer by local official  Faculty, Staff and Participant Introductions **(5 min)**  *Name and institution, why they are attending the training, and role in hospital/health center*  **HBB Objectives (15 min)**  Need for resuscitation in resource-limited countries and overall goal to have person trained at every delivery to help baby breathe. Include global, regional, and local perspectives. |  |
| 9:20 – 9:45AM | **Opening Visualization and Orientation to HBB Materials**  Learner Materials overview (clinical and teaching aids):   - - Learner Workbook   - Facilitator Flipchart   - HBB Action Plan |  |
| 9:45 – 10:00 AM | **Break** |  |
| 10:00-10:45AM | **Preparation for Birth**  Preparation for birth: ***Demonstration*** *of exercise to include mother, birth attendant, helper at front of room;* THEN follow Facilitator Flip Chart [page numbers in brackets]  *Table presentation/demonstration, practice of 4 skills* (3 learner dyads per table):   - Identify helper & review emergency plan [3b] - Prepare area for delivery (clean, warm, well lighted) [3b] - Wash hands [3b] - Prepare area for ventilation & check equipment [3b]   Check yourself questions, exercise and small group discussion |  |
| 10:45 – 11:30AM | **Routine Care**  *Table presentation/demonstration, practice of 5 skills, and check yourself questions:*   - - Dry thoroughly (if meconium is present, clear airway prior to drying) [4b]   - Evaluation: Is the baby crying? [5b]   - Keep warm (skin-to-skin, cover baby with dry blanket & put on hat), check breathing, cut cord, encourage breastfeeding [6b]   - How to clamp or tie and cut umbilical cord [7b]   *Exercise: Routine care and Group Discussion* [8b] |  |
| 11:30AM – 1:00PM | **The Golden Minute, Part I (45min)**  *Table presentation/demonstration, practice, check yourself questions:*   - - Clear airway *if blocked*, stimulate breathing [9b]   - Evaluation: Is baby breathing well? [10b]   *Exercise: The Golden Minute-clear the airway and stimulate breathing and Group Discussion* [11b]  **The Golden Minute, Part II (45 min)**  *Table presentation/demonstration, practice, check yourself questions:*   - Ventilate with bag and mask (initiate ventilation) [12b] - How to ventilate with bag and mask (technique) [13b] - Evaluation: Is the baby breathing well? [14b]   *Exercise: The Golden Minute (including initial steps) part II and Group Discussion* [15b] |  |
| 1-2PM | **Lunch Break** |  |
| 2 – 3PM | **Continued Ventilation with Normal and Slow Heart Rate**  Table presentation/demonstration, practice, check yourself questions:   - Call for help, Improve ventilation [16b] - Is the heart rate normal or slow? [17b] - Ventilate until the baby is breathing well, then monitor with mother [18b] - Continue ventilation and seek advanced care [19b] - Transport mother and baby together, and support the family [20b]   *Exercise: continued ventilation with normal heart rate and Group Discussion* [21b]  *Exercise: continued ventilation with slow heart rate and Group Discussion* [22b] |  |
| 3 – 3:30PM | **Mastering the Action Plan and Preparing for Evaluation**  Practice and scenario development homework (6 cases p. 37 Learner Workbook)  Overview of Evaluation – Knowledge check, bag and mask skills, OSCE A and B  **Preparing for day 2 – Facilitator/Master Trainer Component** |  |
| **Day 2*** | **EVALUATE LEARNERS** |  |
| 8-8:30AM | **Welcome, Review of Day and Plan for Day 2** |  |
| 8:30AM – 10:30AM | **HBB Knowledge and Skills Evaluations**   - Administer HB02 Written Knowledge Check – Multiple Choice Questionnaire (MCQ) - Administer HB03 Bag and Mask Ventilation Skills Performance evaluation - Administer HB04 OSCE-Station A - Administer HB05 OSCE-Station B |  |
| 10:30 – 11AM | **Break** |  |
| 11-11:30AM | Demonstration of how to clean bag, fill and empty NeoNatalie, and pack up equipment |  |
| 11:30AM– 12PM | Review key messages with learners' suggestions recorded as a list of What we will do and What we won't do |  |
| 12 - 12:30PM | Closing Ceremony with certificates and photo (optional) |  |

****Note****: If Day 2 cannot immediately follow Day 1 due to work commitments, Day 2 must be scheduled within one week of Day 1.*

**Global Network Helping Babies Breathe (HBB) Training**

**1C. REFRESHER TRAINING OF THE “ACTIVE” BIRTH ATTENDANTS**

|  | **Pre-Training** |
| --- | --- |
|  | - - - Review goals for the training and course objectives. - Review training agenda in detail: model and review the methods for presentation, demonstration, practice - Set up room: rectangular or square tables of 6, 3 learner dyads per table, and seat for facilitator, Video CD. - On table: one facilitator flip chart, 3 NeoNatalies filled with water, sand timer - Action Plan hanging on walls near tables - Review use of flip charts - Review use of simulators - Plan process for administering pretests (MCQ and OSCE B) and post-tests (MCQ, BMV, OSCE A, and OSCE B) |

|  | **The Training Day** |
| --- | --- |
| 9:00AM -10:00AM | **Arrival, check-in and pre-testing**   - Trainees arrive, complete **pre-test knowledge check** (HB02) - Call one by one for **OSCE B** (HB05) with the Master Trainers - Master Trainer to note the gaps in the knowledge and skills of the trainees. |
| 10:00AM – 10:15AM | **Welcome and introductions**  Discussion about Need for refresher training with the trainees –   - To have person trained at every delivery to help baby breathe - To maintain and reinforce the skills |
| 10:15AM – 10:45AM | **AAP Videos : HBB Refresher Teaching**   - Hand washing - Preparing an area for ventilation and checking equipment - Drying thoroughly - Positioning the head and clearing the airway - Providing stimulation to breathe - Clamping or tying and cutting the cord - Initiating ventilation - Ventilating with bag and mask - Improving ventilation - Evaluating heart rate   **Show videos, narrate script (separate document), stress important points** |
| 10:45 AM – 11:00 AM | **Break** |
| 11:00 AM -11:30AM | **Practice - OSCE A, OSCE B and BMV Skills** |
| 11:30 AM – 12:00Noon | **Monitoring (QA) Activities**   - **Daily Practice**   - Emphasize the need- it will improve the performance & confidence.   - Remind they can do it anytime during the shift, but **daily.**   - Emphasize that they must check **the real life LR equipments daily.**   - They must report defective neonatalies or missing logs immediately.   - Whenever possible, do full OSCE B in the practice.   - Perform practice in pairs where one BA can give a case to the other.   - **Resuscitation Debriefing (RDB)**   - Show a demo (Use the script that is provided)   - Remind that RDB should be done for **every non-macerated baby not crying at birth.** Similarly **HB13** should be filled for all these.   - Remind it should be done **before the shift ends.**   - **RDB must include 3 important things-**     - What went well?     - Next time in similar situation, how could you do better?     - Why this happened? (What maternal/fetal risk factors?) - **Death Audit (DA)**   - Remind that it should be done for **all fresh still births and all deaths within 1 hour of birth including IUDs.** Similarly **HB14** should be filled for all these.   - Remind it should be done **before the shift ends.**   - **DA must include 2 important things-**     - Cause of death     - Other Maternal / fetal risk factors - **Discuss Data Forms (HB10, HB13, HB14) & Show them Graphs** |
| 12:00Noon – 1:30PM | **Post Test**   - **HB02 MCQ Knowledge Test** - **HB03 BMV Test** - **HB04 OSCE-A** - **HB05 OSCE-B** |
